# Catastrophic Kawasaki disease unresponsive to IVIG in a 3-month-old infant: a diagnostic and therapeutic challenge

**DOI:** 10.1186/1546-0096-10-28

**Published:** 2012-08-28

**Authors:** Cristina Medeiros Ribeiro de Magalhães, Natália Ribeiro de Magalhães Alves, Adriana Valença de Melo, Clodoaldo Abreu da Silveira Junior, Yanna Karla de Medeiros Nόbrega, Lenora Gandolfi, Riccardo Pratesi

**Affiliations:** 1 Pediatric Rheumatology Unit, Brasilia General Hospital, Federal District Health Department, Brasilia, DF, Brazil; 2 Graduate Program in Medical Sciences, University of Brasilia School of Medicine, Brasilia, DF, Brazil; 3 Pediatric Research Laboratory, University of Brasilia School of Medicine, Brasilia, DF, Brazil; 4 Department of Pharmaceutical Sciences, School of Health Sciences, University of Brasilia, Brasilia, Brazil; 5 Intensive Care Unit, “Santa Lucia” Hospital, Brasilia, DF, Brazil; 6 Universidade de Brasilia. Faculdade de Medicina, Laboratorio de Pesquisas em Pediatria - Salas B1 94/13, Campus Universitário Darcy Ribeiro, Asa Norte, Brasilia, DF, Brazil

**Keywords:** Kawasaki disease, Catastrophic disease, Refractory Kawasaki, Methotrexate, Etanercept

## Abstract

The present report describes the severe evolution of Kawasaki disease in a three-month-old infant. The ailment was initially atypical in its presentation, with the patient exhibiting only persistent fever in association with a progressive lethargy and maculopapular rash on the face, trunk and limbs erroneously diagnosed as *roseola infantum*. On the 10^th^ day of the condition, mainly due to the unexplained persistence of fever, the infant was admitted to a local hospital. The typical features of KD appeared only on the 14^th^ day of illness with the relapse of the maculopapular rash in association with non-purulent conjunctivitis; dry, reddish and fissured lips; tongue with reddish and hypertrophic papillae; erythema and edema of the palms and soles. During the following days, the ailment rapidly evolved to a catastrophic clinical picture characterized by generalized vasculitis, splenic infarction, pulmonary thrombosis, giant right and left coronary aneurysms, dilatation of common and internal iliac arteries and progressive ischemia of the distal third of the feet resulting in necrotic lesions of both halluces. Appropriate therapy was initiated, but repeated administration of intravenous immunoglobulin G (IVIG) followed by three days of administration of methylprednisolone did not abate the intense inflammatory activity. The remission of inflammation and regression of vascular lesions were only achieved during the following five weeks after the introduction of methotrexate associated with etanercept. The report of this case aims to draw attention to severe forms of KD that exhibit an unfavorable evolution and can be extremely refractory to the conventional therapy.

## Background

Kawasaki disease (KD) is a vasculitis occurring in infants and children characterized by prolonged fever; polymorphous skin rash; erythema of the oral mucosa, lips and tongue; erythema, desquamation and swelling of the palms and soles; bilateral conjunctival injection; and cervical lymphadenopathy. KD is the most common cause of multisystem vasculitis in childhood; although it can cause vasculitis in several organs, such as the lungs, liver, kidneys, intestine, gallbladder and brain, the most commonly damaged are the coronary arteries, frequently resulting in the formation of aneurysms and stenosis. Thus, Kawasaki disease is the leading cause of acquired heart disease in children in developed countries [1].

Despite the numerous theories that have been suggested regarding its etiology, the cause of KD remains unknown. Treatment with intravenous immunoglobulin (IVIG) still persists as the mainstay of KD therapy, reducing the rate of coronary aneurysm formation from 25% to 3-5% [2]. Resistance to IVIG therapy appears to be increasing with reported resistance rates of 20-30% [3].

KD is rare in infants under three months of age. Early diagnosis and treatment of these infants poses a special challenge because they are the least likely to present with signs and symptoms meeting the criteria of persistent fever and at least four of the five other characteristic features. This may result in the delayed initiation of appropriate therapy and may be the reason for the increased frequency of coronary artery abnormalities observed in this age group [4]. In this report, we describe a case of a 3-month-old infant with IVIG and steroid-resistant KD associated with peripheral gangrene, splenic infarction, dilated iliac arteries, pulmonary thrombosis and giant coronary artery aneurysms.

## Case report

A three-month-old girl without a previous history of health problems began to show persisting fever (38°C) that appeared one day after meningococcal vaccination. On the 5^th^ day of continuous fever, a maculopapular rash covering the face, trunk and limbs was observed and considered to be due to *roseola infantum*. The rash subsided after three days, but the infant continued febrile, became progressively lethargic and was admitted for diagnostic investigation on the 10^th^ day of illness. On admission, laboratory investigation showed white blood cell count (WBC) of 25 000/mm^3^ (68% of neutrophil, and 7% of band cells), hemoglobin concentration of 6.5 g/dL, platelet count of 237.000/mm, and serum C-Reactive Protein (CRP) of 19.4 mg/dL (normal value: ≤ 0.5 mg/dL). Chest X-ray imaging was compatible with right upper lobe pneumonia. In view of the child’s lethargy associated with increasing irritability, a lumbar puncture was performed that yielded 142 WBCs (70% of neutrophils), 43 mg/dL of glucose and 134 mg/dL of protein. An intravenous wide-spectrum antibiotic (ceftriaxone) was initiated.

**Figure 1:**
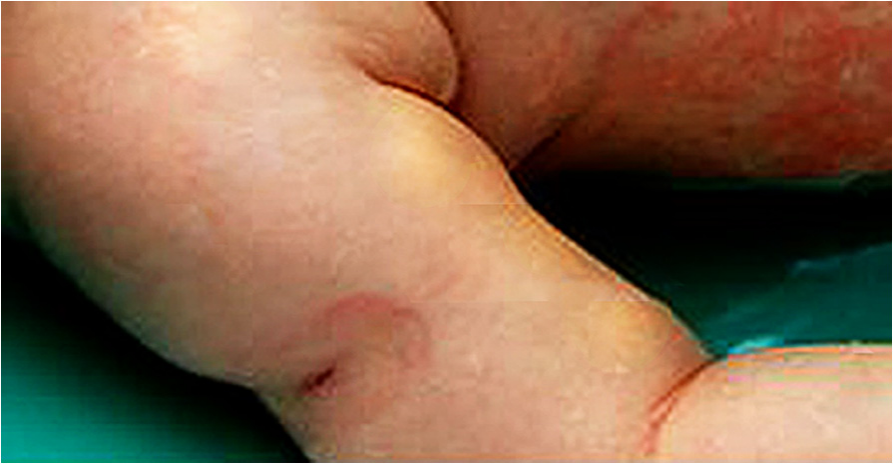
**Erythema and induration at the site of previous vaccination with BCG**.

**Figure 2:**
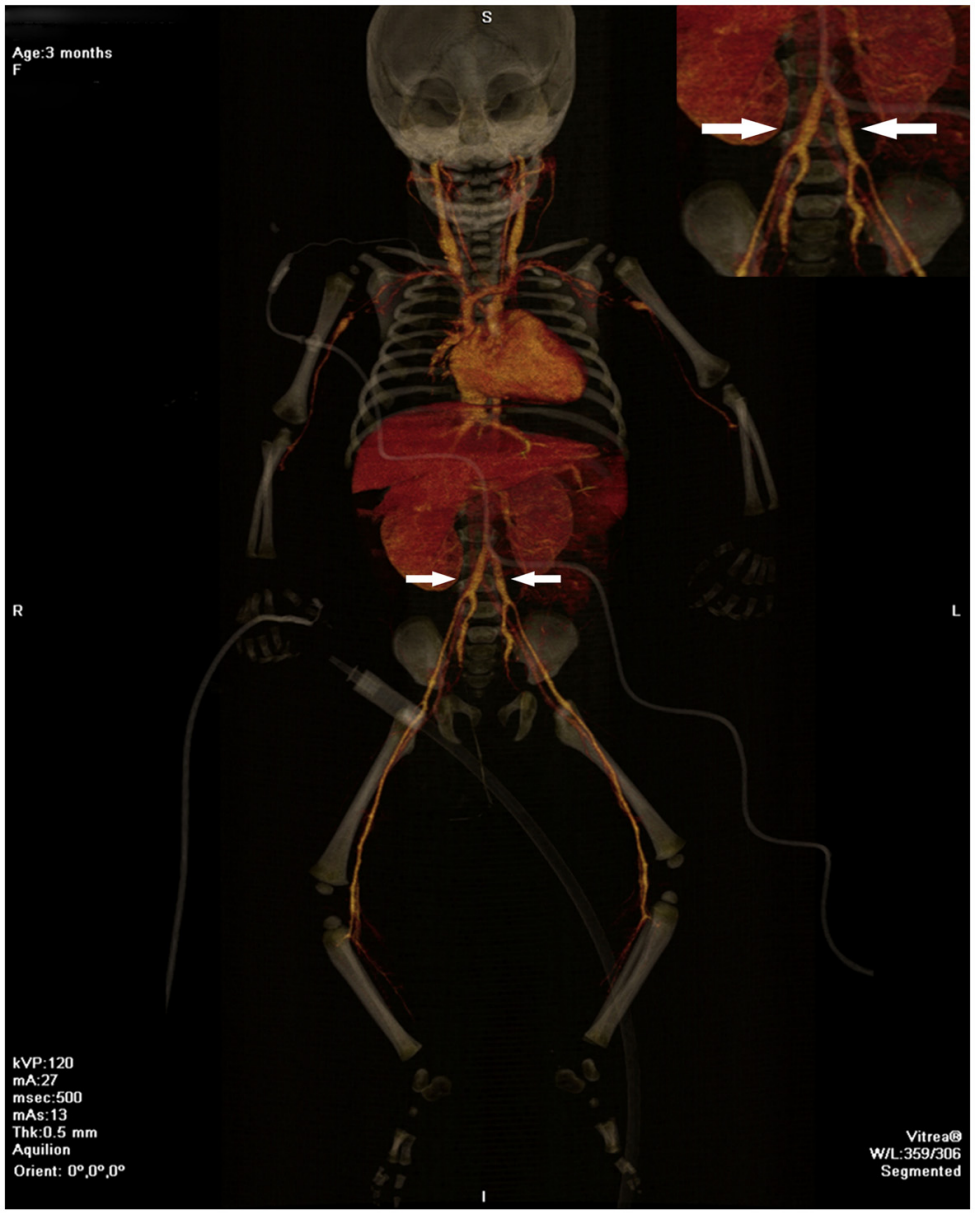
**Whole-body angio-tomography showing the aorta with a normal diameter and significant dilatation of both iliac arteries (see detail in the upper right side of the image)**.

**Figure 3:**
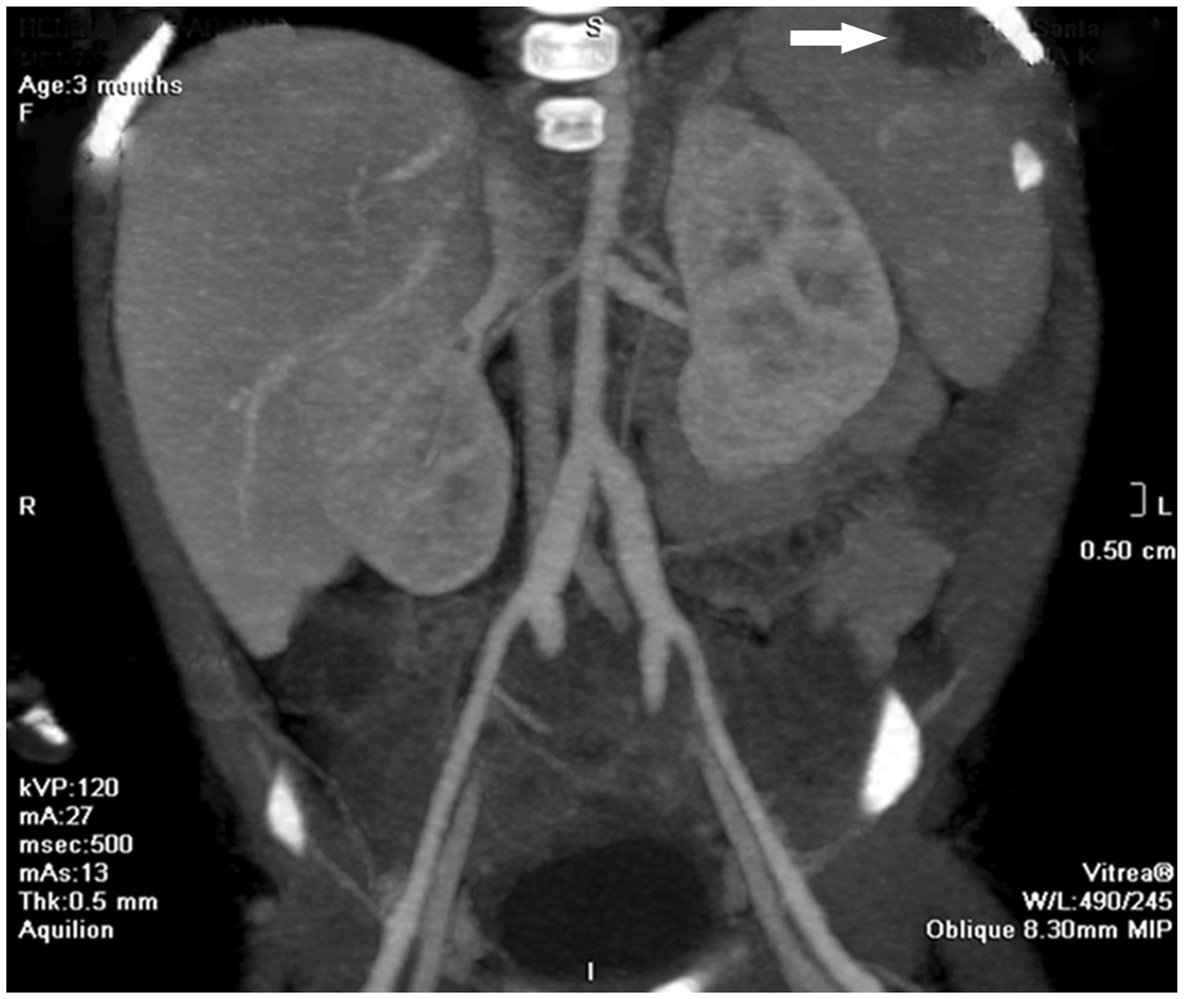
**Whole-body angio-tomography showing area of hypodensity in the splenic parenchyma suggestive of splenic infarction**.

Typical clinical characteristics of KD only appeared in this infant on the 14^th^ day of illness with the relapse of the maculopapular rash in association with bilateral nonpurulent conjunctivitis; dry, reddish and fissured lips; tongue with reddish and hyperthrophic papillae; and erythema and edema of the palms and soles that quickly evolved into a deficit of perfusion, with progressive ischemia of the distal third of the feet. Despite the reintroduction of intravenous wide-spectrum antibiotics (cefepime and vancomycin) and the administration of dopamine, a progressive cyanosis of the toes was observed, followed by hypothermia and blackening of both halluces. Anticoagulant therapy was started with enoxaparin. Inflammation was observed at the site of the BCG vaccination scar (Figure 1) in association with a marked hyperemia of the anal region. An arterial Doppler ultrasound of the lower limbs disclosed an absence of blood flow in the distal tibiofibular branches, and alprostadil IV was started soon after. A computed angio-tomography showed significant dilatation of both iliac arteries (Figure 2), foci of rounded opacities in both lungs suggestive of pulmonary thrombosis, and a hypodense area in the splenic parenchyma suggestive of infarction (Figure 3). At this time a first echocardiography disclosed normal coronary arteries and mild mitral regurgitation. Blood and CSF cultures yielded negative results, but inflammatory markers remained extremely high (erythrocyte sedimentation rate (ESR) 120 mm/h, CRP 34 mg/dL and alpha-1-acid glycoprotein 160 mg/dL). Serologic tests for cytomegalovirus, parvovirus, rubella, and Epstein Barr virus were negative. Soon after the diagnosis was established, a 2 g/kg/dose of intravenous immunoglobulin (IVIG) was administered in association with 80 mg/kg/day of acetylsalicylic acid (ASA) in divided doses. The administration of enoxaparin was suspended two days after the IVIG administration because the ischemic injury of the halluces did not show progression.

On the 17^th^ day of illness, the high fever still persisted in association with significant signs of inflammatory activity. Three days after the administration of this first dose of IVIG, the high inflammatory activity had not subsided (CRP 29.2 mg/dL, alpha-1-acid glycoprotein 143 mg/dl), and a repeated echocardiogram showed moderate pericardial effusion associated with mild mitral regurgitation and significant dilatation (5.2 mm in diameter) of both coronary arteries. The dosage of ASA was decreased to 5 mg/kg/day, as a platelet anti-adhesive agent due to the presence of coronary artery lesions. A single daily dose of 30 mg/kg/day of methylprednisolone IV was started and maintained for three days, followed by prednisolone at a dosage of 2 mg/kg/day, to reduce the inflammatory activity.

In spite of these therapeutic measures the child persisted with fever, inflammatory activity (CRP 8.20 mg /dL) and an increased platelet count (905.000/mm³). Seven days after the first administration, a second 2 g/kg dose of IVIG was given followed by a rapid decline of the fever and improvement of the anal mucositis, the inflammation of the BCG scar and the degree of vasculitis in her feet. A new echocardiogram that was performed eight days after the second IVIG dose showed increase in the previously observed dilatation of the left coronary artery (6 mm) and pericardial effusion. Methylprednisolone IV at a dosage of 30 mg/kg/day was reinitiated and maintained for three days, followed by prednisolone at 2 mg/kg/day to reduce the inflammatory activity.

The infant was finally discharged for follow-up at the walk-in Clinics of Pediatric Rheumatology and Pediatric Cardiology, with the prescription of 5 mg/kg/day of ASA, 2 mg/kg/day of prednisolone and 3.75 mg/kg/day of clopidogrel. Despite her progressive clinical improvement and her normal platelet count (434,000 mm3), 15 days after discharge, she still showed increased inflammatory activity (CRP 10.53 mg/dL, alpha-1-acid glycoprotein 183 mg/dL), total cholesterol (232 mg/dL) and LDL (157 mg/dL). Her echocardiogram showed aneurysms of 5 mm and 7 mm respectively on the right and left coronary arteries, associated with a dilation of 3.5 mm in the anterior descending branch of the left coronary artery. Methotrexate at 0.5 mg/kg /week P.O. and etanercept at 0.8 mg/kg/week administered subcutaneously were added to the drugs already in use.

Five weeks after discharge, the infant was considered to be in clinical remission with inflammatory activity tests, echocardiography, LDL and total cholesterol yielding normal results. On this occasion, a repeated echocardiogram disclosed a significant reduction in the diameter of the aneurysms to 4 mm and 5.5 mm respectively for the right and left coronary arteries, and to 3.2 mm for the anterior descending artery. The child’s condition improved over the next eight months without any clinical or laboratory evidence of inflammatory activity, showing a further regression of her coronary artery abnormalities with the diameters decreasing to 2.4 mm and 3.6 mm respectively for the right and left coronary arteries and to 2.3 mm for the anterior descending artery.

The sequential stages of this infant’ KD evolution are summarized in Table 1.

**Table 1:** Major landmarks in the evolution of Kawasaki disease in this three-year old infant

**Days of evolution**	**Clinical signs**	**Laboratory data**	**Therapy**
Day 1 to 9^th^	Fever; maculopapular rash; lethargy		
10^th^ day	Hospital admission; persisting fever	High CRP; chest X-ray: pneumonia; CSF: aseptic meningitis	Wide spectrum antibiotic (ceftriaxone)
14^th^ day	KD characteristic clinical signs, persisting fever, cyanosis of toes and blackening of halluces, reactivation of BCG scar	Doppler ultrasound: absence of blood flow in distal tibiofibular branches. Computed angio-tomography: dilatation of both iliac arteries; pulmonary thrombosis; splenic infarction. 1^st^ Echocardiogram: normal coronary arteries. High levels of inflammatory markers.	Reintroduction of wide- spectrum antibiotics (cefepime & vancomycin); anticoagulant therapy: enoxaparin; alprostadil; 1^st^ administration of IVIG & ASA (80 mg/kg/day),
17^th^ day	Persisting fever	Inflammatory markers still high; 2^nd^ echocardiogram: significant dilatation of coronary arteries	ASA decreased to (80 mg/kg/day); Started three daily doses of 30 mg/kg/day of methylprednisolone IV followed by 2 mg/kg/day of prednisolone;
21th day	Persisting fever	Inflammatory markers still high;	2^nd^ administration of IVIG
29^th^ day	Fever decline; improvement of feet vasculitis and BCG scar inflammation	3^rd^ echocardiogram: increasing dilatation of left coronary artery; pericardial effusion.	Restarted methylprednisolone IV (30 mg/kg/day), followed by 2 mg/kg/day of prednisolone.
35^th^ day	Hospital discharge	Persisting abnormal levels of inflammatory markers;	Discharge prescription: ASA 5 mg/kg/day and clopidogrel 3.75 mg/kg/day
51^st^ day		Persisting abnormal levels of inflammatory markers; 4^th^ echocardiogram: aneurysms on right and left coronary arteries and dilatation of the descending branch of left coronary artery.	Methotrexate 0.5 mg/kg/week P.O. and etanercept 0.8 mg/kg/week added to drugs already in use
85^th^ day	Considered in clinical remission	Normal results of inflammatory activity tests; 5^th^ echocardiogram : reduction of aneurysm and anterior descending artery dilatation	

## Discussion

This case highlights both the difficulty of reaching a definitive diagnosis in some cases of KD and the severity of this disorder in very young infants. Although KD has been described in different ethnic and racial groups and in all pediatric age groups, its highest incidence is in children under 5 years. Its incidence below six months is relatively rare and even rarer under three months of age. A review of a Japanese database of 105,755 cases reported over a 25-year period yielded only six cases aged 30 days or younger and 1768 cases (1.67%) aged 90 days or younger [5]. Children three months old or younger are more prone to cardiac complications and are frequently affected by an incomplete form of the disease that generally hampers and delays the diagnosis [6].

Another unusual manifestation of this case was that aseptic meningitis was detected as one of the initial manifestations of the disease. Aseptic meningitis has been generally described as an event that occurs during the acute phase of KD [7,8]. Other neurological manifestations such as intense irritability, seizures, ataxia, lethargy or coma, subdural collection, hemiplegia, facial palsy and sensorineural hearing loss can also be detected in approximately 1.3 to 3.7% of cases [9,10]. CNS lesions should always be considered, especially in severe cases accompanied by intense and prolonged inflammatory activity [11].

Aside from the prolonged fever, many of the diagnostic criteria required to establish a definitive diagnosis of KD appeared in this infant only after the 14^th^ day of illness. This led to a delayed diagnosis and delayed treatment with IVIG that should have been administered within the first 10 days of illness. The lack of appropriate therapy with IVIG within the first 10 days in association with prolonged high fever, high inflammatory activity, anemia and hypoalbuminuria in younger infants are all considered predisposing factors for developing coronary aneurysms [1].

This young infant’s disease progression was unusually severe, even for the severe course sometimes seen in infants under six month with KD, and certainly more severe than older children with KD. This age group generally exhibits an increased frequency of coronary damage [12]. The exceptional severity of this case was mainly due to the involvement of various organs (lung, spleen, heart) and several arteries (coronary arteries, anterior descending branch of the left coronary artery and both iliac arteries). The abnormalities of the coronary arteries were exceptionally severe, with their size rapidly increasing to a diameter five times larger than that expected for the patient’s age. In addition to the unfavorable and severe course of the disease, this infant failed to appropriately respond to repeated administrations of 2 g/kg/day of IVIG, and followed by two three-day courses of 30 mg/kg/day methylpredisolone IV and subsequent maintenance dose of 2 mg/kg/day of prednisone. Despite this therapeutic approach, the progression of the inflammatory activity and aneurysms continued unabated.

Approximately15% of patients with KD fail to respond to the first dose of IVIG, and this group generally exhibits a greater risk of coronary abnormalities [13-15]. In those patients in whom a second dose of IVIG did not result in a satisfactory clinical response, the administration of corticosteroids may eventually reduce the degree of inflammatory activity even though it usually does not slow the progression of aneurysms [1,16]. As far as we know, there is no established protocol for those cases in which the above-mentioned measures did not achieve the desired results. Several different therapeutic approaches have been adopted, mostly based on case reports or non-randomized or uncontrolled trials, with apparently favorable outcomes. Immunosuppressive agents such as cyclophosphamide or cyclosporine in association with prednisone or methylprednisolone have been used and resulted in a decrease in fever although they failed to show substantial effects on the progression of coronary abnormalities [17].

Recently, in view of the multiple lines of scientific evidence supporting the prominent role of tumor necrosis factor-α (TNF-α) in mediating inflammation in acute and refractory KD, a new class of biological agents has emerged for the treatment of patients with refractory KD. Monoclonal antibodies to TNF (infliximab) [18] and a soluble form of TNF receptor fusion protein that antagonizes the effects of endogenous TNF (etanercept) showed satisfactory results in several cases of refractory KD leading to a decline in fever and inflammatory activity and to a regression of coronary abnormalities [19,20]. A low dose of methotrexate has also been used to treat patients with resistant KD and has shown promising results with a rapid decrease in fever, improvement in clinical symptoms and normalization of acute phase reactants [20-22].

The treatment in the subacute phase and in convalescent patients with coronary aneurysms is aimed at preventing vessel thrombosis. A low dose of aspirin (3 to 5 mg/kg/day) is the main treatment for children with small and medium-sized aneurysms and may be continued for a prolonged time. Other agents may also be used in association with aspirin such as antiplatelet agents (clopidogrel, ticlopidine, dipyridamole) and show an increased effectiveness in blocking platelet aggregation. Randomized studies are needed to establish the role of agents such as low molecular weight heparin that was used in this case, and warfarin and monoclonal antibodies against the platelet receptor IIb / IIIa in the management of children with giant aneurysms [1].

## Conclusion

In conclusion, the prognosis of KD in infants can be particularly severe, and clinicians should be suspicious of atypical KD in younger infants with persistent fever even if all the criteria for KD are not met. In the present case, despite the adverse clinical progression and imminent risk of infant death, a successful response was finally obtained through combined treatment with methotrexate and etanercept that resulted in a satisfactory remission of the patient’s clinical symptoms within five weeks along with a normalization of laboratory results, associated with a gradual decrease in the diameters of the aneurysms.

## Consent

Written informed consent was obtained from the patient for publication of this report and any accompanying images.

## Competing interests

The authors declare that they have no competing interests.

## Authors’ contributions

CMRM together with NRMA wrote the initial draft of the article and provided expert advice regarding the evolution and treatment of the patient; AVM, CASJ and LG were responsible for follow-up of the patient throughout the course of the disease and compiled and reviewed the results of laboratory tests. RP and YKMN wrote the final version of the paper and were responsible for the responses to the reviewers and the subsequent corrections. All authors read and approved the final manuscript.

## References

[B1] Newburger JW Takahashi M Gerber MA Gewitz MH Tani LY Burns JC Shulman ST Bolger AF Ferrieri P Baltimore RS Wilson WR Baddour LM Levison ME Pallasch TJ Falace DA Taubert KA Committee on Rheumatic Fever, Endocarditis, and Kawasaki Disease, Council on Cardiovascular Disease in the Young, American Heart Association. Diagnosis, treatment, and long-term management of Kawasaki disease: a statement for health professionals from the Committee on Rheumatic Fever, Endocarditis, and Kawasaki Disease, Council on Cardiovascular Disease in the Young, American Heart Association Pediatrics 2004 114 1708 1733 10.1542/peds.2004-2182 15574639

[B2] Newburger JW Takahashi M Beiser AS Burns JC Bastian J Chung KJ A single intravenous infusion of Gama globulin as compared with four infusions in the treatment of acute Kawasaki syndrome N Engl J Med 1991 324 1633 1639 10.1056/NEJM199106063242305 1709446

[B3] Tremoulet AH Best BM Song S Wang S Corinaldesi E Eichenfield JR Martin DD Newburger JW Burns JC Resistance to intravenous immunoglobulin in children with Kawasaki disease J Pediatr 2008 153 117 121 10.1016/j.jpeds.2007.12.021 18571548 PMC2526555

[B4] Pannaraj PS Turner CL Bastian JF Burns JC Failure to diagnose Kawasaki disease at the extremes of the pediatric age range Pediatr Infect Dis J 2004 23 789 791 10.1097/01.inf.0000134312.39744.a4 15295237

[B5] Tsuchida S Yamanaka T Tsuchida R Nakamura Y Yashiro M Yanagawa H Epidemiology of infant Kawasaki disease with a report of youngest neonatal case ever report in Japan Acta Paediatr 1996 85 995 997 10.1111/j.1651-2227.1996.tb14201.x 8863886

[B6] Chuang CH Hsiao MH Chiu CH Huang YC Lin TY Kawasaki disease in infants three months of age or younger J Micribiol Immunol Infect 2006 39 387 391 17066200

[B7] Türel O Güzeltaş A Aydoğmuş C Hatipoğlu N Hatipoğlu H Siraneci R Kawasaki disease presenting as meningitis in a two months old infant Anadolu Kardiyol Derg 2011 11 369 370 21592935 10.5152/akd.2011.091

[B8] Dengler LD Capparelli EV Bastian JF Bradley DJ Glode MP Santa S Newburger JW Baker AL Matsubara T Burns JC Cerebrospinal fluid profile in patients with acute Kawasaki disease Pediatric Infect Dis J 1998 17 475 478 10.1097/00006454-199806000-00008 9655538

[B9] Terasawa K Ichinose E Matsuishi T Kato H Neurological complications in Kawasaki disease Brain Dev 1983 5 371 374 10.1016/S0387-7604(83)80041-2 6638393

[B10] Tabarki B Mahdhaoui A Selmi H Yacoub M Essoussi AS Kawasaki disease with predominant central nervous system involvement Pediatr Neurol 2001 25 239 241 10.1016/S0887-8994(01)00290-9 11587880

[B11] Fujiwara S Yamano T Hattori M Fujiseki Y Shimada M Asymptomatic cerebral infarction in Kawasaki disease Pediatr Neurol 1992 8 235 236 10.1016/0887-8994(92)90077-C 1622525

[B12] Chang FY Hwang B Chen SJ Lee PC Meng CC Lu JH Characteristics of Kawasaki disease in infants younger than six months of age Pediatr Infect Dis J 2006 25 241 244 10.1097/01.inf.0000202067.50975.90 16511387

[B13] Durongpisitkul K Soongswang J Laohaprasitiporn D Nana A Prachuabmoh C Kangkagate C Immunoglobulin failure and retreatment in Kawasaki disease Pediatr Cardiol 2003 24 145 148 10.1007/s00246-002-0216-2 12457253

[B14] Wallace CA French JW Kahn SJ Sherry DD Initial intravenous gammaglobulin treatment failure in Kawasaki disease Pediatrics 2000 105 e78 10.1542/peds.105.6.e78 10835091

[B15] Freeman AF Shulman ST Refractory Kawasaki disease Pediatric Infect Dis J 2004 23 463 10.1097/01.inf.0000125893.66941.e0 15131473

[B16] Shulman ST Is there a role for corticosteroids in Kawasaki disease? J Pediatr 2003 142 601 603 10.1067/mpd.2003.258 12838185

[B17] Pinna GS Kafezis DA Tselkas OI Skevaki CL Kawasaki disease: an overview Curr Opin Infect Dis 2008 21 263 270 10.1097/QCO.0b013e3282fbf9cd 18448971

[B18] Oishi T Fujieda M Shiraishi T Ono M Inoue K Takahashi A Ogura H Wakiguchi H Infliximab treatment for refractory Kawasaki disease with coronary artery aneurysm Circ J 2008 72 850 852 10.1253/circj.72.850 18441471

[B19] Choueiter NF Olson AK Shen DD Portman MA Prospective Open-Label Trial of Etanercept as Adjunctive Therapy for Kawasaki Disease J Pediatr 2010 157 960 966 10.1016/j.jpeds.2010.06.014 20667551 PMC2970727

[B20] Lee TJ Kim KH Chun JH Kim DS Low-dose Methotrexate Therapy for Intravenous Immunoglobulin-resistant Kawasaki Disease Yonsei Med J 2008 49 714 718 10.3349/ymj.2008.49.5.714 18972590 PMC2615375

[B21] Ahn SY Kim DS Treatment of intravenous immunoglobulin-resistant Kawasaki disease treated with methotrexate Scand J Rheumatol 2005 34 136 139 16095010 10.1080/03009740510026328

[B22] Lee MS Anh SY Jang GC Kim DS A case of intravenous immunoglobulin-resistant Kawasaki disease treated with methotrexate Yonsei Med J 2002 43 527 532 12205742 10.3349/ymj.2002.43.4.527

